# Deep Neural Networks for Simultaneously Capturing Public Topics and Sentiments During a Pandemic: Application on a COVID-19 Tweet Data Set

**DOI:** 10.2196/34306

**Published:** 2022-05-25

**Authors:** Adrien Boukobza, Anita Burgun, Bertrand Roudier, Rosy Tsopra

**Affiliations:** 1 Université Paris Cité Sorbonne Université Inserm, Centre de Recherche des Cordeliers Paris France; 2 Inria HeKA PariSanté Campus Paris France; 3 Department of Medical Informatics Assistance Publique – Hôpitaux de Paris Hôpital Européen Georges-Pompidou Paris France; 4 ESIEE Cité Descartes Noisy le Grand Cedex France

**Keywords:** neural network, deep learning, COVID-19, explainable artificial intelligence, decision support, natural language processing

## Abstract

**Background:**

Public engagement is a key element for mitigating pandemics, and a good understanding of public opinion could help to encourage the successful adoption of public health measures by the population. In past years, deep learning has been increasingly applied to the analysis of text from social networks. However, most of the developed approaches can only capture topics or sentiments alone but not both together.

**Objective:**

Here, we aimed to develop a new approach, based on deep neural networks, for simultaneously capturing public topics and sentiments and applied it to tweets sent just after the announcement of the COVID-19 pandemic by the World Health Organization (WHO).

**Methods:**

A total of 1,386,496 tweets were collected, preprocessed, and split with a ratio of 80:20 into training and validation sets, respectively. We combined lexicons and convolutional neural networks to improve sentiment prediction. The trained model achieved an overall accuracy of 81% and a precision of 82% and was able to capture simultaneously the weighted words associated with a predicted sentiment intensity score. These outputs were then visualized via an interactive and customizable web interface based on a word cloud representation. Using word cloud analysis, we captured the main topics for extreme positive and negative sentiment intensity scores.

**Results:**

In reaction to the announcement of the pandemic by the WHO, 6 negative and 5 positive topics were discussed on Twitter. Twitter users seemed to be worried about the international situation, economic consequences, and medical situation. Conversely, they seemed to be satisfied with the commitment of medical and social workers and with the collaboration between people.

**Conclusions:**

We propose a new method based on deep neural networks for simultaneously extracting public topics and sentiments from tweets. This method could be helpful for monitoring public opinion during crises such as pandemics.

## Introduction

### Background

Pandemics caused by emerging pathogens are public health emergencies. They have dramatic consequences for the population (mortality, morbidity, social life) and the economy [1]. The number of outbreaks has increased in recent decades, and this trend is expected to intensify [1] in the next years. In particular, when the first cases of pneumonia caused by the SARS-CoV-2 pathogen were declared in Wuhan, Hubei Province, China [2,3], the virus rapidly spread around the world, leading the World Health Organization (WHO) to declare a pandemic on March 11, 2020, and announced it on Twitter with the tweet: “BREAKING “We have therefore made the assessment that #COVID19 can be characterized as a pandemic”-@DrTedros #coronavirus.” With this declaration occurring on social media, Twitter remains an ideal medium to study public opinion on the declaration of the COVID pandemic.

### Utility of Social Networks for Identifying Sentiments and Topics of the Population During Pandemics

As public engagement is a key element for mitigating pandemics [4-6], several studies have already mined social media since the beginning of the COVID-19 pandemic but with distinct objectives (eg, infoveillance) [7-9] or during different periods (eg, when first important measures were taken in the United States) [7,10-14]. To our knowledge, there is no study analyzing public opinion in the immediate reaction just after the WHO announcement.

Social networks have largely been used to capture public opinion, especially during outbreaks (eg, Ebola [15], H1N1 [16]). The methods used to analyze texts from social networks have considerably improved over time: manual analysis first, followed by natural language processing (NLP) approaches based on syntactic-semantic or statistical techniques [17], and more recently, deep learning approaches [18,19]. Deep learning methods provide new perspectives on text analysis since they give the possibility to (1) integrate semantic information around text (eg, with pretrained word embedding, which allows higher semantic information as the input for the neural network rather than a one-hot encoder [20]) and (2) analyze a significantly larger corpus of text nearly in real time, making it possible to discover new evidence faster [21]. These approaches [7,8,22-26] have already been used to capture topics (eg, for the Covid Infoveillance study [7] or insulin pricing concerns in the United States [27]) or sentiments (eg, on social network posts or on health care tweets [17,28-30]).

### Prior Work With Topic Extraction

Several approaches have been used for topic extraction, including qualitative analysis, descriptive analysis, and topic analysis.

#### Qualitative Analysis

Qualitative analyses [22,23,31] capture common themes from manual analysis, fragmentation, and labelling of text. This method has demonstrated its capacity to accurately capture new and complex topics [32] but with some major issues: It requires human coders, time, and resource consumption and is not suitable for use with high-dimensional data.

#### Descriptive Analysis

Descriptive analyses [8] capture the distribution of word frequencies by studying the repetition of words among topics identified from the internet. It allows researchers to correlate the importance of a topic to the volume of searches among this peculiar topic. The main pitfall of this method is the inability to consider the context around the word.

#### Topic Analysis

Topic analysis is a method used to discover topics that occur in a collection of documents and has largely been used to mine social media. This method aims at identifying patterns in documents using NLP approaches. Two main categories of topic analyses are commonly used: topic classification [33] and topic modeling [34].

Topic classification uses supervised learning algorithms (eg, Naïve Bayes [19], support vector machine [SVM] [35]) that need to be trained beforehand with labeled documents, consequently requiring a priori knowledge of corpus topics. These algorithms can achieve variable performance, with a precision varying from 44.9% to 93.3% [19], depending on the methods used.

On the contrary, topic modeling uses unsupervised learning algorithms that do not need to be trained beforehand. They are thus less work-intensive than supervised learning algorithms since they do not need human-labelled data but often require larger data sets and are less precise than supervised learning algorithms. Latent semantic analysis is the traditional method for topic modeling [36]. It is based on the distributional hypothesis and assumes that words with close meaning will occur in similar pieces of text [37]. This assumption enabled the development of algorithms such as latent Dirichlet allocation (LDA) [7,25,26,38], which is popular in the medical domain [39]. This algorithm identifies latent topics from words tending to occur together and outputs *n* clusters of words grouped together by similarity. The topics are then manually labelled according to the interpretation of the set of words within each cluster [7,40]. However, LDA requires the investigator to predefine the number of topics and does not consider the sequence of words [39]. Topic modeling has been poorly assessed, perhaps a result of the difficulty comparing the clusters obtained with a gold standard. To overcome this lack of evidence, Zhang et al [38] proposed an original approach for assessing LDA: They compared the topics extracted from LDA to those collected through a national questionnaire survey and reported a kappa concordance coefficient of 0.72.

### Prior Work With Sentiment Analysis

Several approaches have been used for sentiment analysis, including lexicon-based methods, supervised machine learning methods, and hybrid methods.

#### Lexicon-Based Methods

Lexicon-based methods are unsupervised methods that do not require training an algorithm and depend only on existing dictionaries [29]. These methods assume that the polarity of a text (positive or negative) can be obtained by characterizing the constituent words within [29]. A key argument for their adoption was the fact that they only compute the number of positive and negative words [41] and thus are faster to implement. They are also easily adaptable to various languages by using language-specific dictionaries [42]. However, they present some limitations that come with language analysis, especially regarding negation, sarcasm, or words with different meaning [28,29]. Furthermore, they are essentially limited by the size, coverage, and quality of the dictionary [17]. Interestingly, lexicon-based methods can achieve an accuracy up to 94.6% [43], depending on the dictionary used [43-46].

#### Supervised Machine Learning Methods

Supervised machine learning methods, which require time to be trained, have also been used [47]. Naïve Bayes often better operates on well-shaped data, whereas SVM often achieves better results with low-shaped data. As social media are poor-quality data, due to very varying length of tweets, colloquial language, and numerous spelling mistakes, larger training data sets are needed to achieve good performance, and the complexity of these methods may impact training time [48]. They can achieve variable performance, with reported accuracies ranging from 48% to 91% [47,49,50], depending on the algorithm used.

#### Hybrid Approaches

Hybrid approaches combine both previous methods. In a recent literature review, Drus and Khalid [29] demonstrated that hybridized approaches to sentiment analysis often outperform lexicon-based or machine learning–based approaches alone. For example, Hassan et al [47] used lexicon annotation and multinomial Naïve Bayes for depression measurement from social networks and reported an accuracy rate of 91%; Zhang et al [51] used lexicon annotation and SVM to annotate sentiments from tweets and reported an accuracy of 85.4%.

### Prior Work Aiming to Capture Both Topics and Sentiments

Few methods based on topic-sentiment models have been developed, including the joint sentiment topic (JST) model, Topic-Sentiment Mixture (TSM) model, and Time-aware Topic Sentiment (TTTS) model.

#### Joint Sentiment Topic Model

The JST [52] model is a probabilistic modelling framework that extends LDA with a new sentiment layer. JST is fully unsupervised and extracts both topics and sentiments at a document level [52]. However, JST ignores the word ordering (bigrams or trigrams [52]). Reverse JST [53] is derived from JST with an inversion of the order of the topic and sentiment layers. The Aspect and Sentiment Unification Model (ASUM) [54] is close to JST but focuses on the sentence level. These models have been poorly assessed and were essentially applied on nonmedical data sets, with an accuracy varying from 59.8% to 84.9% for JST [52,53] and 69.5% to 75.0% for reverse JST [53].

#### Topic-Sentiment Mixture Model

TSM [55] is based on the probabilistic latent semantic indexing model and includes an extra background component and 2 sentiment subtopics. It has been assessed on various weblog data sets [55] but suffers from problems of inferencing on new documents and overfitting data [52] and requires postprocessing to obtain the sentiment [56].

#### Time-Aware Topic Sentiment Model

More recently, the TTTS model [57] is a joint model for topic-sentiment evolution, based on LDA and allowing analysis of topic-sentiment evolution over time [57].

### Strengths and Weaknesses of Previous Work

Many approaches have proven useful for identifying public topics alone but without the associated sentiment. Other works, especially hybrid approaches, have proven useful for sentiment detection alone but cannot capture the topics alongside sentiment detection.

In both cases, this makes the results less informative and useful [52]. Simultaneously capturing topics and sentiments would be more relevant for better comprehension of public opinion [52], especially in a time of crisis. Topic-sentiment models have been proposed for the simultaneous capture of public opinion and sentiments but may require prior domain knowledge and have not been applied yet to the medical and social media domains [52,53,55].

### Potential for a Neural Network–Based Approach to Advance This Area of Research

Neural networks have achieved impressive performances in many NLP tasks, such as sentiment prediction [58-60]. Furthermore, the probabilities generated by neural networks could be used to represent sentiment intensity through a quantitative scale leading to more precise information than basic sentiment classification into dual qualitative classes (negative or positive). Surprisingly, to our knowledge, they have not been used yet for the simultaneous capture of public topics and sentiments from social media.

Here, we propose incorporating convolutional neural networks (CNNs) in conjunction with sentiment lexica to simultaneously capture public topics and sentiments in a hybridized approach [18,29]. The simultaneous capture of public topics and sentiments, without prior knowledge, would be very useful during crises, such as the COVID-19 outbreak.

## Methods

### Preparation of the Tweet Data Set for Use as an Input for Neural Networks

#### Data Collection

To analyze the immediate effect of the announcement of the COVID-19 pandemic by the WHO, we focused on tweets relating to coronavirus posted on Twitter the day after the announcement. We collected all tweets containing the keywords “coronavirus” or “COVID” posted in English as recognized by Twitter services on March 12, 2020 (ie, from 00:00:01 to 23:59:59). For each tweet, we extracted the tweet ID, text content, and time stamp. We also filtered them using the language parameter of Twint Python Library [61] to allow the extraction of English-written tweets only. We verified the absence of tweets in other language by using common stop words of these languages, resulting in only finding foreign city names or family names.

We extracted 1,386,496 tweets from Twitter’s database with the Twint Python library and stored them in the JSON format.

### Ethical Approval

Ethic approval was not needed as analysis of large bodies of text written by humans on the internet and in some social media such as Twitter (eg, quantitative analysis such as infodemiology or infoveillance studies or for qualitative analysis) is not considered “human subjects research.”

#### Data Preprocessing

We removed 241,506 (17.5%) duplicate tweets and retweets to limit the risk of overrepresentation of one person’s view. Twitter elements (URLs, links to pictures, hashtags, mentions), punctuation, isolated letters, and typographic UTF-8 characters, such as stylized commas or apostrophes, were also removed. Likewise, stop words from Porter’s list [62] were removed using the Python library Natural Language Toolkit (NLTK) [63], with orthographic variations. Tweet content was then lower-cased, and “coronavirus” and “COVID” were mapped under a unique term.

**Figure 1:**
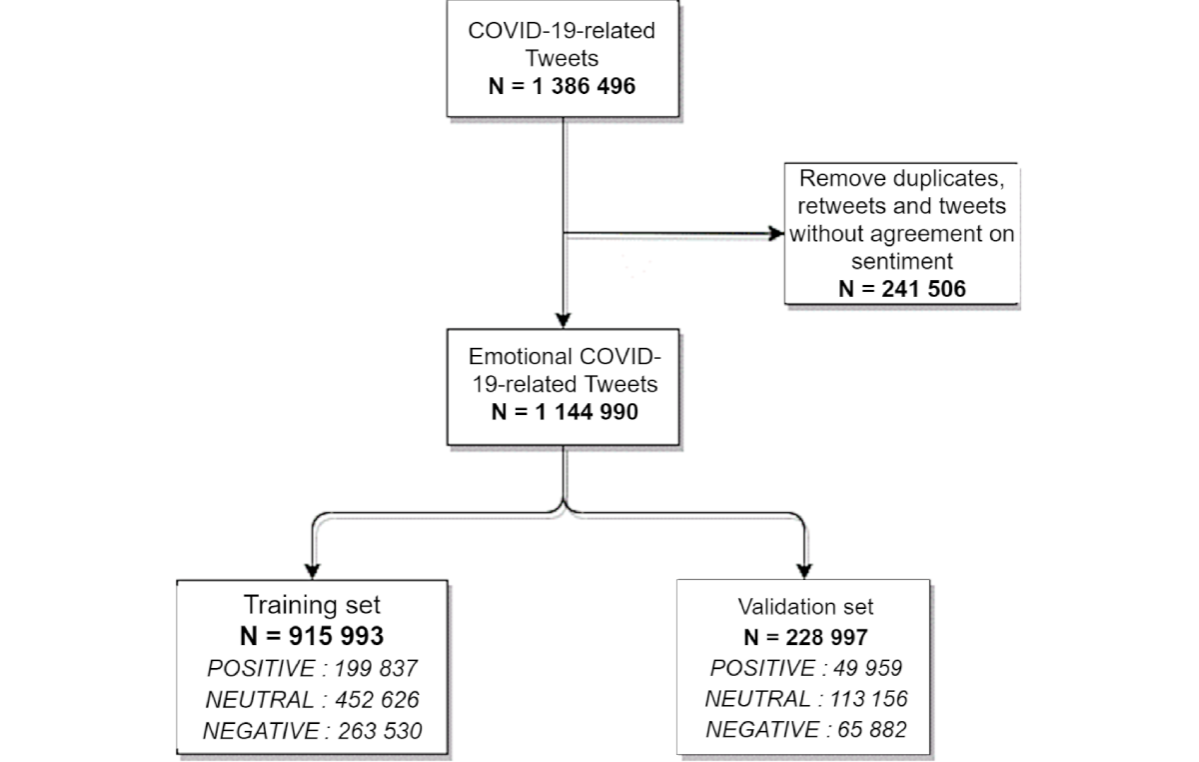
Study flowchart.

Figure 1 provides a flow chart of tweet collection, preprocessing, and splitting into the training and testing sets.

#### Sentiment Annotation

Each tweet was automatically annotated with 3 sentiment labels from 3 different sentiment lexicons from R package tidytext [64] (AFINN [44], BING [43], and NRC [45,46]). These lexicons have largely been used in previous works [30,42,44]. Each lexicon provided a numerical value for each sentiment word in the tweet, and these values were summed to annotate the general sentiment of the tweet for each lexicon considered, as described in other works [41,42]. Thus, for each annotation, the sum value could be positive, equal to 0, or negative resulting in positive, neutral, or negative annotation by the considered sentiment lexicon.

Annotation conflicts were handled using a simple rule-based algorithm to compute a single annotation for each tweet. This algorithm is based on the majority vote method and produced a unique qualitative annotation as “positive,” “neutral,” or “negative.” If a majority vote was not obtained (ie, if each algorithm returned a different statement), the tweets were excluded from the data set.

The automatic annotation of included tweets was controlled on 50 randomized tweets, using a manual revision of tweet annotation, resulting in an overall agreement of 86% between algorithm and manual annotation, resulting in a kappa coefficient score of 0.73.

### Deep Neural Networks for Simultaneously Capturing Public Topics and Sentiments

#### Tokenization, Word Embedding, and CNN Architecture

CNN architecture was chosen as it is known to consider Ngrams, making various levels of analysis possible.

All words in each tweet were tokenized, and tweets were postpadded for use as input into the pretrained embedding layer of the neural networks, which encoded semantic properties for each token. We used a 25-dimension Global Vector for word representation (GloVe) embedding trained on 2 billion tweets to shorten training time and achieve better results. This embedding is available from the GloVe project page [65].

The resulting vectors were then passed to a convolutional unit composed of a convolutional layer (able to analyze unigrams, bigrams, or trigrams), global max pooling layer, dense layer, and dropout layer for regularization and prevention of overfitting. A final dense layer composed of 3 units alongside a softmax activation function computed the probabilities of the tweet belonging to each class of sentiment (positive, neutral, negative). Early stopping was used to prevent overfitting when training our models.

To perform the supervised learning step, the data set was split using stratification over sentiment annotation, allocating 80% (915,993 tweets) for training and 20% for validation (228,997 tweets; Figure 1). The best model was found after 10 training iterations and used a kernel size of 2 on the convolutional layer. The accuracy was 81%, and the F1 score was 81% on the validation data set (Table 1).

**Table 1:** Performance of the neural network for sentiment prediction.

Performance measure	Positive	Neutral	Negative	Total
Accuracy	83%	80%	82%	81%
F1 score	79%	82%	81%	81%
Precision	77%	85%	79%	82%
Recall	82%	80%	83%	81%

#### Neural Network Outputs: Sentiment Intensity Score and Weighted Word Capture

For each tweet, we captured the dominant sentiment as a sentiment intensity score that was calculated from the 3 probabilities predicted by the CNN:

SIS = P(POSITIVE) x 1 + P(NEUTRAL) x 0 + P(NEGATIVE) x (–1)

where SIS, P(POSITIVE), P(NEUTRAL), and P(NEGATIVE) are sentiment intensity score and probabilities for a tweet to belong to the positive, neutral, and negative sentiment classes, respectively, according to the neural network.

Applying this formula allowed us to distinguish 21.82% (249,796/1,144,990) of the tweets as positive, 49.41% (565,782/1,144,990) as neutral, and 28.77% (329,412/1,144,990) as negative. The sentiment intensity score of each tweet was then represented on a scale from –100% (totally negative) to +100% (totally positive), permitted by using the softmax activation function.

**Figure 2:**
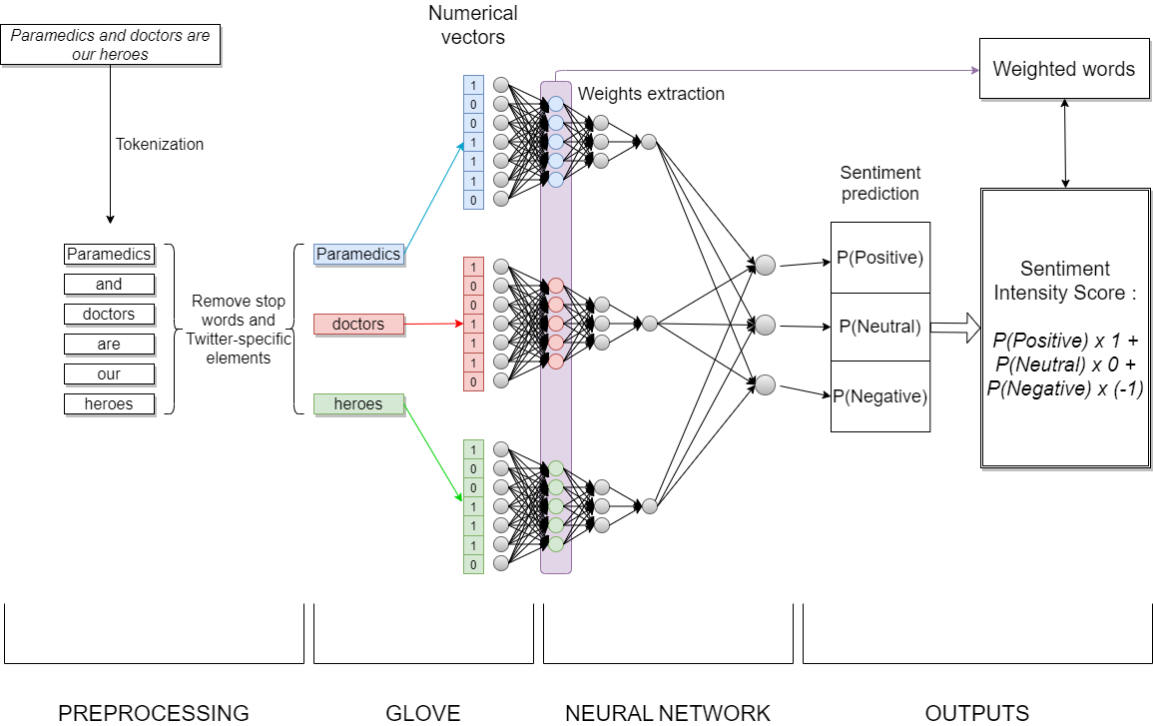
Neural network outputs, where P(POSITIVE), P(NEUTRAL), and P(NEGATIVE) are the probabilities for a tweet to belong to the positive, neutral, and negative sentiment classes, respectively, according to the neural network. Please note that the convolutional neural network (CNN) is represented here as a simple perceptron to facilitate reading, and each word’s contribution score is represented with colored neurons.

As the CNN architecture alternates convolutional and pooling layers, it allows, first, aggregation of the numerical input coming from each word separately until a hidden layer and then combination of the values of this hidden layer until the output of the CNN. Hence, this hidden layer encompasses a value for each word, and this value can be seen as a contribution score (or a weight) of each word in the computation of the final output of the CNN [66]. As the output of the CNN is used to compute the dominant sentiment intensity of the whole tweet, the intermediate values extracted from the hidden layers make it possible to associate “weighted words” to the sentiment intensity score of the tweet. Figure 2 summarizes the capture of the sentiment intensity score and of the weighted words.

In previous steps, the weighted words and sentiment intensity score were captured at the individual tweet level. At the tweet data set level, we computed the average weight of each word for each sentiment intensity score by gathering similar words from distinct tweets and applying a mean function. The resulting matrix contained the weighted words for each given sentiment intensity score.

#### Visualization of Neural Network Outputs

We developed a Shiny [67] application (available at [68]) based on word cloud representation to visualize the weighted words for each sentiment intensity score. This application provides 2 panels: On the right panel, the word cloud displays the weighted words for a given sentiment intensity score. On the left panel, the word cloud can be customized through options specifying the sentiment intensity score, the number and type of words to display (coronavirus or sentiment-related terms), and the esthetics (eg, palette of colors, total percentage of vertical words, and use of a radial gradient).

To generate our word clouds, we replaced the use of word frequencies to summarize text documents by the weights calculated in our matrix. The visualization was made clearer by grouping all lexical variants of a word together, using the word lemmatizer from the R package textstem [69]. We also implemented options allowing the user to ignore all sentiment words and emojis, to choose the word count threshold for display, and to choose the precision of the sentiment score (integer or float to 1 or 2 decimal places).

#### Identification of the Main Topics Discussed by the Public and Their Associated Sentiment Intensity

Using the Shiny interface, we captured the highest weighted words for the most extreme sentiment intensity scores (negative sentiment: –100; positive sentiment: +100). Author A Boukobza then manually analyzed the top 100 words for both extreme sentiments using string-matching techniques and identified main negative and positive topics within tweets. Each topic was assigned by the manual analysis of these words. Then, we calculated the number of tweets discussing each topic within the data set.

In the results section, we replaced the real names of politicians, political parties, websites, and media with anonymous epithets such as “politicianX,” “politicalPartyX,” “webX,” “mediaX.”

**Figure 3:**
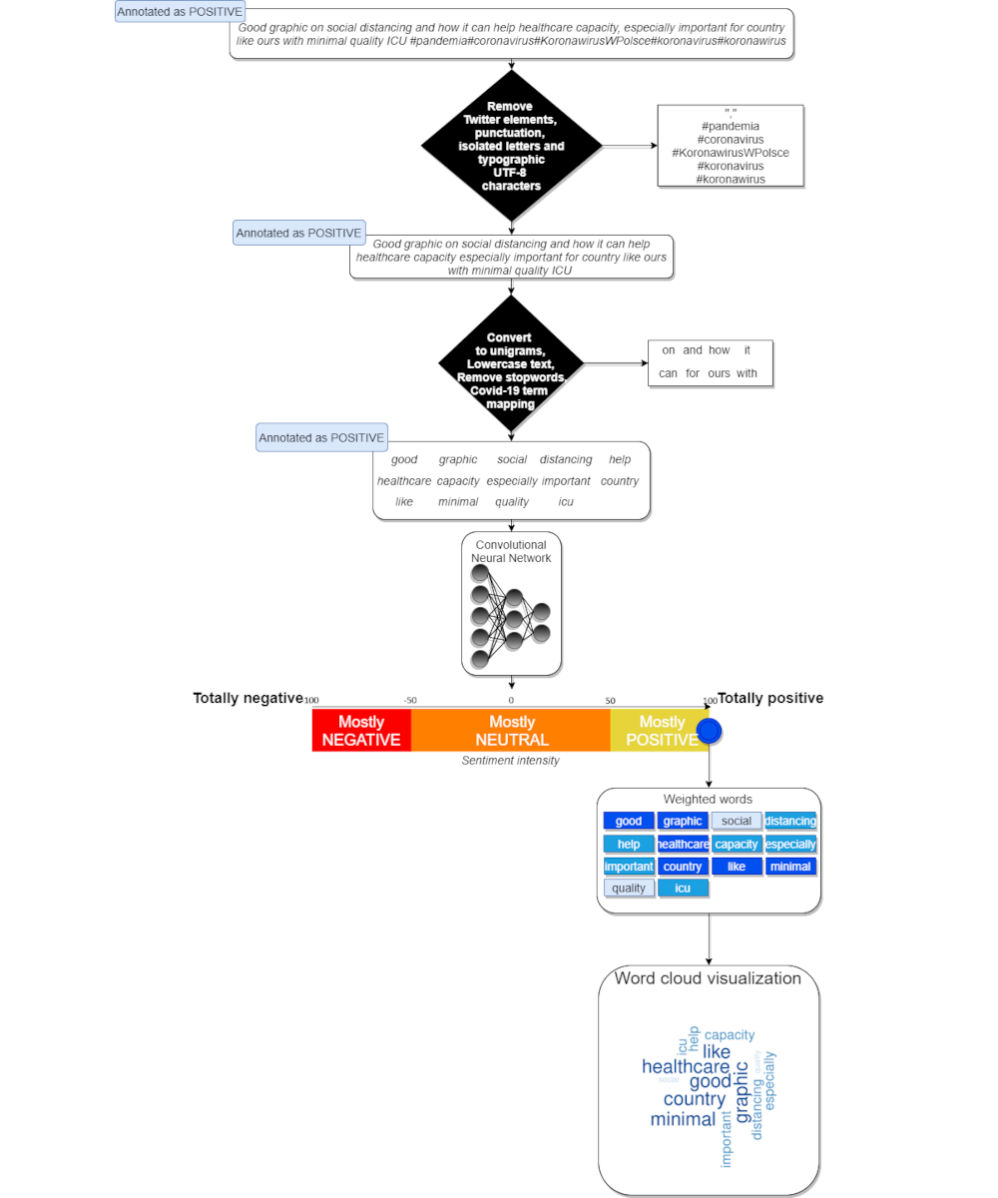
Method used for simultaneously extracting weighted words and their associated sentiments from tweets. An example of a tweet at each step is provided, from initial preprocessing to sentiment intensity scale classification (here, the tweet sentiment score is +100%) and final output as a word cloud.

Figure 3 summarizes the general method used for extracting weighted words and their associated sentiments from Twitter data.

## Results

### Visualization of Neural Network Outputs With an Interactive Interface

Neural network outputs were visualized with an interactive interface displaying a word cloud composed of the weighted words for each sentiment intensity score.

**Figure 4:**
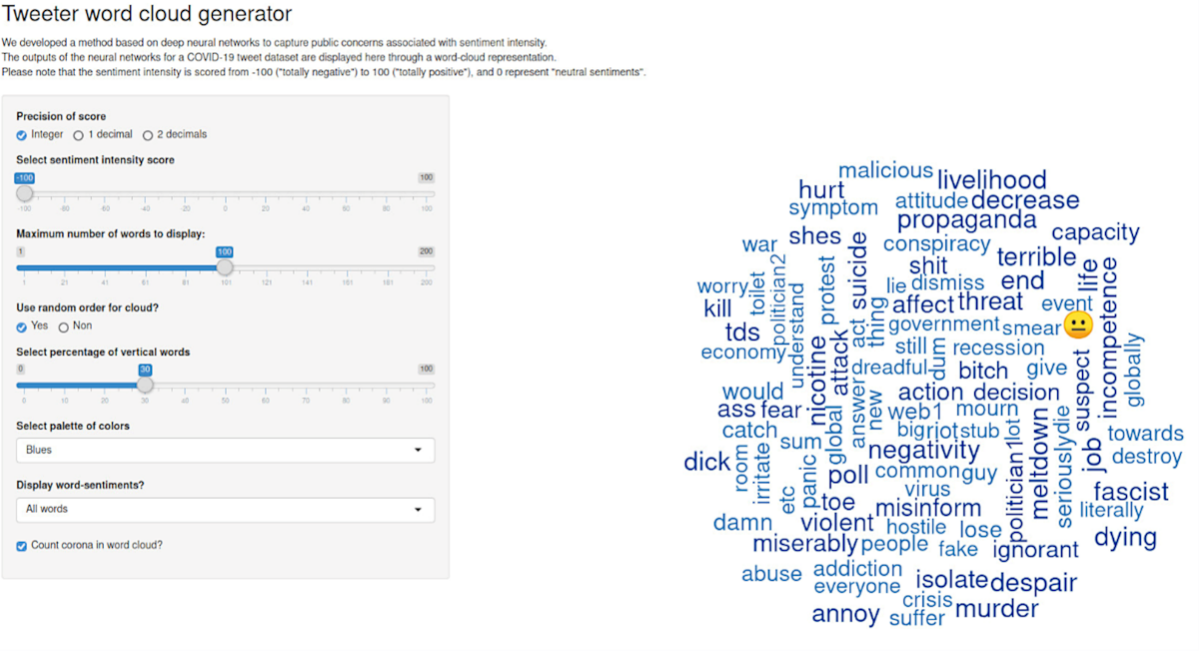
Interactive web application for visualizing neural network outputs. The real names of politicians, political parties, websites, and media were replaced by anonymous epithets such as “politicianX,” “politicalPartyX,” “webX,” “mediaX.”.

The analysis of the top 100 most important words for each class allowed us to predistinguish main themes retrieved for positive, negative, and neutral classes. In the totally positive class (ie, +100 sentiment intensity score), the top 100 words included words such as “happiness,” “democratic,” “ethical,” “quarantine,” or “expertise.” Concerning the neutral class (ie, 0 sentiment intensity score), the top 100 words included names (eg, “François,” “Eliott”), adverbs (eg, “thankfully,” “formally”), or scientific words (eg, “petri,” “aneurysm”). In the totally negative class (ie, –100 sentiment intensity score), the top 100 words included words such as “job,” “economy,” “afraid,” “panic” (Figure 4).

### Identification of Public Topics and Associated Sentiment Intensity

Using word cloud analysis, we captured the topics for both extreme positive and negative sentiment intensity scores that were discussed in Twitter in immediate reaction to the announcement of the pandemic by the WHO. The analysis of these topics revealed that public opinion was extremely negative about the consequences of the pandemic on the economy and health care system. Conversely, public opinion was extremely positive regarding the mutual aid and cooperation between people and the public health measures taken against the spread of COVID-19. More details are given in the following sections, and example tweets are provided in Table 2.

**Table 2:** Main positive and negative topics, with highest weighted words, illustrative tweets, and the number of tweets containing the weighted word.

ID			Topics		Weighted words identified by the neural network		Example of an original tweet		Number of tweets containing weighted words for each topic
**Negative topics**									
	1	International situation		italian, china, eu, euro, italy, politican1, politicalParty1, politicalParty2, politician2, president, government, politician3, incompetence, fascist		*Italy* is already today worse affected by Covid-19 than *China*. (...)		11,297	
	2	Economy		job, impact, industry, yougov, hire, financial, market, livelihood, diarrhea, recession, economy		(...) The *markets* are trash, every *industry* is freaking out, and people are losing their *jobs* because it’s stalling the *economy* and no one is *hiring*. (...)		3486	
	3	Media and social media		media1, media2, american		Signed out of my *media1*. (...) *Media2* is a HORRIBLE thing to be on with this damn Coronavirus (...)		1428	
	4	Media and social media		media1, media2, american		Can the media be declared enemies of the people? They (...) lie to us, (...) and fail to report news/statistics that *Americans* need to know. (...)			
	5	Medical situation		ventilator, paramedic, triage, ration, supply		The (...) most dreadful thing we might face is *rationing* or *triaging* who gets *ventilators*. Emergency rooms across the U.S (...) have limited capacity and *supplies* (...)		1411	
	6	Public health measures		stay, senior, travel, indoor, cancel, ban		The EU *travel* *ban* (...) I must admit is terrible decision extremely terrible (...)		8396	
	7	COVID-19 origin		coronavirusHoax, fake, conspiracy, propaganda		(...) the *Fake* News Media are fabricating the hype and panic to destroy the economy (...) #Pandumbic #*coronavirusHoax*		1680	
**Positive topics**									
	8	International situation		italy, nhs, democracy, gov, politician4		(...) Freer and more *democracy* countries can do this if they take needed measures.		2178	
	9	Economy		client, colleague, customer, company		We would like to extend our heartfelt appreciation to all of our *clients* and partners working on the front-lines (...)		745	
	10	Medical situation		mask, research, health, healthy, resources, healthcare, doctor, applause, hero		Put all your money and *resources* into getting the cure for the Coronavirus you look like a hero and win the election		4803	
	11	Public health measures		stay, control, announce, interpersonal, family, canceleverything, relative, country, precaution, sanitation, icu, measures, prevention, protect		Good graphic on social distancing and how it can help healthcare capacity, especially important for a *country* like ours with minimal quality ICU #pandemia #coronavirus #KoronawirusWPolsce #koronavirus #koronawirus		6642	
	12	Mutual aid and cooperation		collaborative, together		(...) Communities who work *together* to ensure the health and well-being of their fellow neighbor will be stronger and healthier than those who don’t. #Coronavirus		470	

### The 6 Main Negative Public Topics Discussed on Twitter in Immediate Reaction to the Announcement of the Pandemic by the WHO

Regarding the international situation, Twitter users were worried about the situation in Italy (eg, the number of cases exceeding those in China; Table 2, ID 1) or the risk of punishment or imprisonment for Italians not respecting lockdown. They also discussed travel bans and their consequences, such as the US decision to ban all flights to Europe at a time at which only Italy had a major COVID-19 epidemic. Crisis management and decisions taken by politicians, such as decisions relating to paramedical staff management, were also highly criticized. Regarding economy, Twitter users expressed their fears about the economic consequences of COVID-19. They were worried about the shortages induced by panic buying, such as those leading to a shortage of toilet rolls, and anxiety about the possibility of losing their jobs and being unable to pay their debts (Table 2, ID 2). They also mentioned a potential global recession crisis, caused partly by flight limitations. Regarding media and social media, Twitter users were angry with the media and social media, which they blamed for amplifying fears and stress relating to COVID-19 (Table 2, ID 3), and for not reporting COVID-19 statistics (Table 2, ID 4). Regarding the medical situation, Twitter users were concerned about the medical situation, particularly the management of paramedical staff and materials. They expressed worries about the small number of ventilators available and the likely consequences in terms of equality of access to health care (Table 2, ID 5). Regarding public health measures, Twitter users complained about the limitations of personal liberties, such as the prohibition of flights to Europe (Table 2, ID 6) and the canceling of many events. Regarding the COVID-19 origin, Twitter users talked about “CoronavirusHoax.” They suggested that the pandemic was a hoax and that COVID-19 was a fake disease and evoked a conspiracy theory driven by economic and political motives (Table 2, ID 7).

### The 5 Main Positive Public Topics Discussed on Twitter in Immediate Reaction to the Announcement of the Pandemic by the WHO

Regarding the international situation, Twitter users expressed their satisfaction with the actions and decisions taken by some countries, such as Japan, Hong Kong, Singapore, South Korea (Table 2, ID 8), or Denmark (eg, the decision to impose a lockdown at the right timing). They also highlighted the efficient measures taken by some countries such as the United Kingdom to overcome the negative effects of lockdown (eg, National Health Service access or online courses for students). Regarding the economy, Twitter users were very grateful to all those who worked during the crisis (Table 2, ID 9). Public workers were even described as “people working hard for ensuring population security.” Twitter users were also informed about the continuity of services ensured by some private companies despite the crisis. They were satisfied with the health measures taken by these companies (eg, social distancing, sanitizing measures, provision of masks). Regarding the medical situation, Twitter users maintained their trust and hope regarding the medical situation. They highly appreciated the work of medical and paramedical staff and their involvement in communicating reliable information about COVID-19 to the population. They highlighted the importance of developing telemedicine and evoked the possibility of a COVID-19 vaccine and its potential consequences for health policies (Table 2, ID 10). They also discussed the production and free distribution of infographics and masks to health professionals by private companies. Regarding public health measures, Twitter users encouraged the respect of national measures, social distancing, and lockdowns to allow people to protect themselves and their families. They also appreciated the graphics providing guidance on the changes in behavior required to limit the spread of coronavirus (Table 2, ID 11). Regarding mutual aid and cooperation, Twitter users were satisfied with the level of cooperation between people in front of the coronavirus crisis (Table 2, ID 12). They were grateful to workers and medical and paramedical staff.

## Discussion

### Principal Findings

We proposed here an original new approach based on deep neural networks for the simultaneous capture of public topics and sentiments from Twitter data. We trained a CNN on a training data set of 915,993 tweets and achieved a performance of 81% for both accuracy and F1 score. The trained neural network was able to capture the weighted words and their associated sentiment intensity score. These outputs were then visualized through an interactive and customizable web interface displaying the weighted words as a word cloud representation. The trained model was then used to analyze public topics and sentiments in reaction to the announcement of the COVID-19 pandemic by the WHO.

### Strengths and Limitations

Our study has several strengths. We combined lexicons and deep learning approaches to improve sentiment prediction. We used CNN to capture simultaneously weighted words associated with sentiment intensity score and to compare unigrams, bigrams, and trigrams during training. We also tried to improve the explicability of the model and to limit the black box effect [70,71] by displaying the outputs of the neural networks through an interactive word cloud interface. The word cloud representation is easily understandable and made it possible to consider the outputs attributed by the neural networks to each word according to sentiment intensity score. Our study has also several limitations. First, our method was developed on a data set of tweets in English and needs to be adapted for other languages [72] and assessed with other extensive data sets [49,73]. Another limitation is the finite set of inclusion keywords, resulting in a potential lack of information due to the total number of keywords used. Further works should concentrate on the diversification of keywords used to provide better sensibility. Furthermore, duplicate tweets and retweets were removed during preprocessing to limit the risk of overrepresenting one person’s view, but this may have also led to underestimating the weights of some words. Second, class imbalance was checked before training, and early stopping was used to prevent the neural network from overfitting the data set. This resulted in good performance, with a model accuracy of 81%. Published studies have reported accuracies ranging from 48% to 91% [47,49,50] with the use of supervised learning techniques such as SVM, Naïve Bayes, logistic regression, or word2vec models. However, these performances were measured for binary sentiment classification (ie, negative vs positive sentiment). Here, we decided to consider neutral sentiments too, because it has been shown that tweets can be associated with neutral sentiments [74]. This choice allowed us to give more explicability and granularity but remains an issue because of our inability to compare our results with those of other studies.

### Comparison With Prior Work

#### Use of Social Media to Capture Public Opinion

Approaches other than social media mining have been described. Focus groups provide a good understanding of public opinion and sentiments but are time-consuming and not necessarily representative of the whole population [4,6,75] as shown by Rowe et al [76] during the avian influenza crisis. Telephone and web-based surveys are expensive and time-consuming [77]. Systematic reviews analyze studies capturing public opinion [75] but are inappropriate in pandemic conditions as they require multiple skill sets (eg, experts on the topic, systematic review methodologists) and are hardly usable for real-time monitoring. Unlike these approaches, social media mining captures a large range of opinions from a large sample, rapidly and for a reasonable cost [38,75]. It also has proven useful for understanding the attitudes and behavior of the public during a crisis [78]. For example, before the COVID pandemic, Chew et al [16] used Twitter to extract public perceptions of H1N1 during the H1N1 pandemic. However, some limitations are inherent to social media: The studied population is limited to social media users [79], the geographic location of users cannot be assumed with absolute certainty [80], and analyses are limited to a given language and source (eg, Twitter). Our study illustrates that, despite these issues, social media mining remains an efficient way to capture the thoughts, feelings, and fears of part of the population during a pandemic.

#### Research Perspectives

As the detection of topics and sentiments is directly related to neural network accuracy, more options could be explored to obtain higher scores, such as replacing word2vec embedding with Embeddings from Language Models (ELMo) [75] or Bidirectional Encoder Representation from Transformers (BERT) [14], which have proven useful for aspect-based sentiment classification [4,76]. The development of a Twitter-specific version of sentiment lexicons integrating web-specific elements such as emojis, abbreviations, or hashtags might also improve results [77]. Future research should concentrate on adding more granularity to the emotion expressed in tweets, by using emotion-specific lexicons to annotate the tweets with specific emotions such as fear, sadness, or happiness [21]. Newly developed initiatives such as the Linguistic Inquiry and Word Count (LIWC) dictionary [81] could also fulfill this task as they provide a dictionary able to recognize emotional words and automatically categorize them as more granular emotions in a hierarchical way (ie, each granular emotion, such as anger, is a child of a top-level emotion like a negative emotion).

#### Implications for Public Health

Our method could be used to guide public health decisions [77]. Besides factual parameters such as the disease characteristics or the burden it poses to the health care system [77], public opinion must also be considered to ensure that public health decisions are in line with the beliefs and priorities of the public [77]. Since many people use social media to share opinions and sentiments [79], they could provide policy makers and clinicians an opportunity to understand, in real time, the expectations, beliefs, and behaviors of the population and to adapt public health decisions accordingly [82,83]. They can also be used to communicate timely messages to the population [84] and thus to increase the chance of successful adoption of measures by the population. The development of indicators based on the real-time tracking of health-related conversations on social media is becoming crucial [9,85-87]. A major contribution of this study is to show the usefulness of deep learning methods to simultaneously capture public opinion and associated sentiments from large amounts of social media data.

### Conclusions

We developed a new approach to conduct both sentiment and topic analyses on social media data by leveraging deep neural networks in conjunction with lexicons. We visualized the outputs of the neural network through a word cloud web interface displaying the weighted words associated with each sentiment intensity score. We demonstrated the utility of our method by applying it to a COVID-19 data set and identifying the main positive and negative topics discussed on Twitter in reaction to the announcement of the pandemic by the WHO. Future studies should concentrate on improving neural network performance and adding granularity to emotion detection. Our method may eventually prove useful for developing indicators for monitoring public opinion during pandemics.

## Abbreviations

ASUM: Aspect and Sentiment Unification Model
BERT: Bidirectional Encoder Representation from Transformers
CNN: convolutional neural network
ELMo: Embeddings from Language Models
GloVe: Global Vector for word representation
JST: joint sentiment topic
LDA: latent Dirichlet allocation
LIWC: Linguistic Inquiry and Word Count
NLP: natural language processing
NLTK: Natural Language Toolkit
SVM: support vector machine
TSM: Topic-Sentiment Mixture
TTTS: Time-aware Topic Sentiment
WHO: World Health Organization

## Data Availability

The data that support the findings of this study are not openly available due to Twitter identifying information and are available from the corresponding author upon reasonable request.
